# Evaluation of the Hypoglycemic Potential of Leaves Extract of *Spondias pinnata* (L.f.) Kurz. from Nepal

**DOI:** 10.1155/2021/3230351

**Published:** 2021-06-25

**Authors:** Kusum Sai, Sumit Bahadur Baruwal Chhetri, Shankar Raj Devkota, Deepa Khatri

**Affiliations:** ^1^Department of Pharmaceutical Sciences, School of Health and Allied Sciences, Pokhara University, Pokhara 33700, Nepal; ^2^Department of Biochemistry and Molecular Biology, Monash University, Clayton, VIC 3800, Australia

## Abstract

*Spondias pinnata* (L.f.) Kurz. (family: Anacardiaceae) is a wild deciduous tree indigenous to southeast Asian countries. Different parts of this plant are used traditionally for the treatment and cure of various disorders and illnesses. *S. pinnata* leaves are used to prevent and treat diabetes in traditional Balinese medicine. However, scientific study on the antihyperglycemic effect of its leaves has not been reported yet. Therefore, this study aims to perform phytochemical screening and investigate the hypoglycemic potential of *S. pinnata* leaves extract. Preliminary phytochemical screening of the hydroethanolic extract was performed following the standard tests. *In vivo* hypoglycemic activity of the leaves extract was evaluated using normal and glucose-loaded rats. The results displayed the presence of phytochemical constituents such as saponins, phenolic compounds, flavonoids, and terpenoids. *S. pinnata* (500 mg/kg) and metformin (100 mg/kg) exhibited a significant (*p* < 0.05) decrease in blood glucose level at 1, 2, and 3 h in normal rats when compared to the control group. Metformin- (100 mg/kg)- and *S. pinnata-* (500 mg/kg)- treated groups showed a maximum decrease in the blood glucose level at 3 h after single-dose administration in the oral glucose tolerance test (OGTT). In conclusion, *S. pinnata* leaves possess a significant hypoglycemic activity in the animal model and thus support its traditional use to treat diabetes. Therefore, a detailed mechanism-based study and isolation of bioactive compounds from *S. pinnata* leaves would be beneficial in the future for the search of new hypoglycemic agents.

## 1. Introduction

Diabetes mellitus is a metabolic disorder characterized by hyperglycemia caused due to defects in insulin production, insulin sensitivity, or both. The major complications of diabetes include abnormally high blood sugar levels and blood vessel diseases, which may further cause long-term damage to vital organs such as the eye, kidney, nerves, and heart [1]. In recent years, the prevalence of diabetes has increased worldwide. The global prevalence of diabetes is estimated to be 9.3% in 2019, rising to 10.2% by 2030 and 10.9% by 2045 [2]. The International Diabetes Federation (IDF) has reported that approximately 463 million people (20–79 years) are living with diabetes in 2019, and the number is projected to rise to 578 million by 2030 and 700 million by 2045. Diabetes has caused around 4.2 million deaths worldwide [3]. Treatment and management of diabetes include a healthy diet, physical exercise, medications to lower blood sugar levels, and insulin therapy [4]. Inhibition of enzymes involved in the decomposition of carbohydrates such as *α*-amylase and *α*-glucosidase is an important therapeutic approach for reducing postprandial hyperglycemia [5]. Treatment of diabetes with insulin and other synthetic drugs is associated with various side effects. Therefore, searching for more effective and safer hypoglycemic drugs is still going on all over the world.

**Figure 1:**
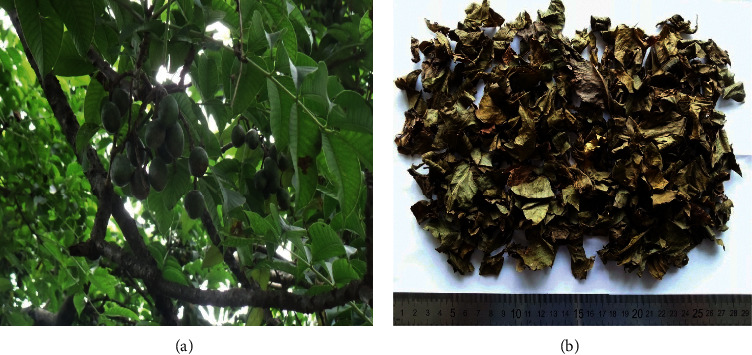
(a) Leaves and fruits of *S. pinnata*. (b) Dried leaves of *S. pinnata*.

*Spondias pinnata* (L.f.) Kurz. (Anacardiaceae) (Figure 1), commonly known as hog plum or wild mango, is a deciduous tree native to Malesia and distributed throughout India, Nepal, Bhutan, Southern China, and Myanmar [6, 7]. The plant bears edible fruits which are eaten fresh or pickled [8]. Different parts of *S. pinnata* are used for medicinal purposes by indigenous people. In Balinese ethnobotanical tradition, the leaves are used to prepare an herbal drink called “Loloh” to treat urolithiasis, heartburn, and diabetes and to boost overall body health [9]. The bark is used for treating dysentery, muscular rheumatism, and diabetes mellitus in Ayurvedic medicine [10]. Similarly, bark juice is given for diarrhea, dysentery, stomachache, and rheumatism in hilly regions of Nepal [8]. Flowers are used in curry and for flavoring in Illam, Nepal [11]. Plant latex is applied for treating wounds and cuts in far-west Nepal [12]. *S. pinnata* possesses various chemical constituents. Its unripe fruit contains methyl caffeate and rhamnetin-3-*O*-sophoroside [13]; the bark contains methyl gallate [14]; the aerial part contains stigmast-4-en-3-one, *β*-sitosterol, and *β*-sitosterol *β*-D-glucoside [15]. Hypoglycemic activity of *S. pinnata* bark has been reported previously [16]. Likewise, antidiabetic and antilipidemic properties of the fruit extract have also been reported [17]. The leaves extract of *S. pinnata* possess antimicrobial and antiviral properties and contain a large number of phenolic compounds exhibiting free radical scavenging ability [18].

However, the hypoglycemic activity of *S. pinnata* leaves has not been documented yet. Therefore, for the first time, the hypoglycemic potential of leaves extract was investigated in normal and glucose-loaded rats as a preliminary effort to reveal its antihyperglycemic properties.

## 2. Materials and Methods

### 2.1. Chemicals and Reagents

Ferric chloride hexahydrate (FeCl_3_.6H_2_O), potassium iodide, iodine, mercuric chloride, and picric acid were purchased from Sigma-Aldrich, USA. Ethanol, hydrochloric acid, sulphuric acid, chloroform, benzene, ammonia, and sodium hydroxide were procured from Merck, Germany, and sodium chloride and gelatin were purchased from Qualigens Fine Chemicals, India. The drugs metformin and glucose were obtained from Times Pharmaceuticals, Nepal. All chemicals used were of analytical grade.

### 2.2. Plant Material and Extraction

Fresh leaves of *S. pinnata* were collected from Kaski district, Gandaki province, Nepal, and identified with the help of locals and using literature [8]. The voucher specimen (PUCD-2018-07) was authenticated by botanist Dr Radheshyam Kayastha and was deposited in the Pharmacognosy laboratory, Pokhara University, for future reference. Dried leaves (30 g) were extracted twice with 80% ethanol in the ratio of 1 : 8 for 24 h at room temperature in a closed vessel with occasional shaking. The filtered extracts were then dried in a rotary evaporator (Heidolph, Germany) to obtain a viscous mass and stored at 4°C until use. The percentage extract yield value was calculated using the following equation:

(1) $$\begin{array}{c} \text{yield}\left(\%\right)=\left(\frac{\text{weight of extract}}{\text{weight of dried leaves}}\right)\times 100. \end{array}$$

### 2.3. Phytochemical Screening

The hydroethanolic extract of *S. pinnata* leaves was tested for potential chemical constituents through preliminary phytochemical screening using standard methods [19, 20].

### 2.4. Experimental Animals

Male albino Wistar rats of 50–60 g were purchased from Pokhara, Nepal, and were housed in polypropylene cages under standard laboratory conditions. The temperature of the facility was maintained at 25 ± 3°C, humidity 55 ± 5%, and light/darkness alternated 12 h apart. Rats were allowed to grow and acclimatize in the laboratory condition for 6 weeks and fed with standard diet and water *ad libitum*. After acclimatization, experiments were performed in rats weighing 150–180 g. They were fasted overnight before the experiment, allowing free access to water.

### 2.5. Ethical Clearance

Ethical clearance to conduct the *in vivo* study was obtained from the Institutional Review Committee (IRC) of the Pokhara University Research Center (ref. no.:148-074-075). All activities using animals were conducted following the ethical guidelines.

### 2.6. Acute Toxicity Study

The acute toxicity of *S. pinnata* leaves extract was determined using male albino Wistar rats (150–180 g) maintained under standard conditions. Overnight fasted rats were taken and divided into four groups of five animals each (*n* = 5). Different doses (250, 500, 1000, and 2000 mg/kg body weight) of the extract were administered orally. Then, the animals were observed for general signs of toxicity for 14 days [21].

### 2.7. Experimental Protocol for *In Vivo* Hypoglycemic Activity

#### 2.7.1. Hypoglycemic Effect in Normal Rats

Rats were kept fasting overnight with free access to water and were divided into four groups, each containing five animals (*n* = 5). Group I was treated with normal saline (0.9% NaCl) and served as a normal control group. Groups II and III were treated with *S. pinnata* leaves extract at the doses of 250 mg/kg and 500 mg/kg body weight, respectively, and Group IV was treated with standard drug metformin (100 mg/kg body weight). Fasting blood glucose levels were determined at the beginning of the experiment. After the oral administration of the test samples, blood glucose levels were measured at 0.5, 1, 2, and 3 h with the help of a clinical glucometer.

#### 2.7.2. Hypoglycemic Effect in Glucose-Induced Hyperglycemic Rats (OGTT)

Oral glucose tolerance test (OGTT) was performed in overnight-fasted normal rats [22] with some modifications. Rats were randomly selected and divided into four groups (*n* = 5). Group I served as a normal control group treated with normal saline (0.9% NaCl). Groups II and III were treated with *S. pinnata* leaves extract at 250 mg/kg and 500 mg/kg body weight, respectively, and Group IV was treated with the standard drug metformin (100 mg/kg body weight). After 30 min, glucose was administered orally to rats of all the groups (2 g/kg body weight). Blood samples were collected at 0, 0.5, 1, 2, and 3 h of glucose administration, and glucose levels were estimated using a clinical glucometer.

### 2.8. Statistical Analysis

Results are expressed as mean ± standard deviation (SD). The *in vivo* data were analyzed using one-way ANOVA, followed by Dunnett's post hoc test to compare blood glucose levels between control and test groups. A *p* value less than 0.05 was considered statistically significant. The statistical analysis was carried out using the Statistical Package for the Social Sciences (SPSS) 20.0 version.

## 3. Results

### 3.1. Phytochemical Screening

Phytochemical investigation of the hydroethanolic leaves extract of *S. pinnata* revealed secondary metabolites such as saponins, phenolic compounds, flavonoids, and terpenoids (Table 1). The extract yield value was found to be 32.14% as calculated.

### 3.2. Acute Toxicity Study

The maximum dose of 2000 mg/kg produced no mortality in rats. The animals did not manifest any signs of restlessness, respiratory distress, irritation, coma, or convulsions. Hence, the doses 250 and 500 mg/kg of *S. pinnata* leaves extract were considered safe for experimental purposes.

### 3.3. *In Vivo* Hypoglycemic Activity

#### 3.3.1. Hypoglycemic Effect in Normal Rats

The effects of two different doses of *S. pinnata* leaves extract on blood glucose levels in normal rats were assessed, and the results are depicted in Table 2. Fasting blood glucose levels were within the range of 94.6–102.6 mg/dl in all groups at 0 h. Both *S. pinnata* and metformin significantly reduced the fasting blood glucose levels in normal rats. *S. pinnata*- (250 mg/kg)- treated group showed a distinct reduction in the blood glucose level after 3 h of extract administration. In contrast, the group treated with 500 mg/kg and metformin (100 mg/kg) significantly reduced blood glucose levels at 1, 2, and 3 h after single-dose administration when compared to the control group.

#### 3.3.2. Hypoglycemic Effect in Glucose-Induced Hyperglycemic Rats (OGTT)

Experimental induction of hyperglycemia by oral administration of glucose (2 g/kg) resulted in increased blood glucose levels after 30 min of glucose administration in all groups (Table 3). Fasting blood glucose levels were within the range of 92.8–98.6 mg/dl in all groups initially. *S. pinnata*- (500 mg/kg)- and metformin- (100 mg/kg)- treated groups showed a maximum decrease in blood glucose levels at 3 h after single-dose administration when compared to glucose levels at 0 h. *S. pinnata* (250 mg/kg) showed a relatively lower reduction in the blood glucose level at 3 h. Therefore, the high dose (500 mg/kg) was more effective than the low dose (250 mg/kg) to lower the blood glucose level.

## 4. Discussion

Diabetes mellitus, popularly known as diabetes, is a global health problem and one of the leading causes of death worldwide. The latest data show that, around 463 million adults are surviving with diabetes [3]. Controlling blood glucose levels is an essential intervention for treating and managing diabetes and related complications [23]. Until now, diabetes is being controlled with medications including allopathic, homeopathic, and traditional medicines [24]. Plant-based natural products have been used as a source of drugs since ancient times [25]. Research studies have shown that plants contain various bioactive compounds with known pharmacological activities [26, 27]. Different traditional medicine systems and folklore medicines utilize medicinal plants as a therapeutic aid for the management of diabetes [28]. For instance, *Momordica charantia, Terminalia chebula, Acacia arabica, Eugenia jambolana, Allium cepa, Aloe vera,* and *Tinospora cordifolia* have been widely used in crude forms and formulations for the treatment of diabetes in Ayurveda [29, 30].

In this study, the *in vivo* hypoglycemic activity of the hydroethanolic extract of *S. pinnata* leaves was investigated in normal and glucose-loaded hyperglycemic rats. *S. pinnata* leaves extract (500 mg/kg) and metformin (100 mg/kg) significantly reduced the blood glucose levels in normal rats at 1, 2, and 3 h on single-dose administration (Table 2). The results of the oral glucose tolerance test (OGTT) showed that metformin (100 mg/kg) and *S. pinnata* (500 mg/kg) reduced blood glucose level maximum at 3 h on glucose- (2 g/kg)- loaded rats (Table 3). However, *S. pinnata* (250 mg/kg) was not able to improve glucose tolerance up to 2 h when compared to the fasting blood glucose levels. The significant reduction in blood glucose level observed may be due to the presence of phytochemical constituents that contribute to its hypoglycemic effect. Preliminary phytochemical screening of *S. pinnata* leaves extract revealed the presence of phenolics, flavonoids, saponins, and terpenoids, which may be responsible for its hypoglycemic action. Zheng et al. [31] reported that total saponins identified from Chinese medicinal plants significantly reduced fasting blood glucose and serum insulin levels and also eliminated oxidative stress in experimental rats. Terpenoids isolated from some antidiabetic medicinal plants have been found to stimulate insulin secretion from *β*-cells or mimic insulin action [32]. Steroids demonstrated significant antidiabetic activity by reducing the high blood glucose level and restoring insulin levels in streptozotocin-induced diabetic rats [33]. The possible mechanism by which plant extracts show antidiabetic activity may be attributed to bioactive compounds that may increase insulin secretion or its release from unbound forms [34]. In addition to the potent phytochemical constituents, it has strong free radical scavenging and moderate *α-*amylase inhibitory activities [35]. *α*-Amylase is an enzyme responsible for the digestion of carbohydrates. It hydrolyses *α-*linked polysaccharides such as starch and glycogen and converts them into simple sugars such as glucose and maltose [36]. Inhibition of carbohydrate digesting enzymes delays glucose absorption and is thus a critical therapeutic approach for reducing postprandial hyperglycemia [5]. Hazra et al. [37] found that *S. pinnata* leaves and stem bark have high antioxidant and free radical scavenging activities and contain a large amount of flavonoids and phenolic compounds [37]. Flavonoids such as catechins improve glucose uptake by regeneration of damaged beta cells. Similarly, epicatechin gallate, epigallocatechin gallate, and epigallocatechin facilitate glucose mobility by activating transporters [24, 38]. Thus, the hypoglycemic activity of *S. pinnata* leaves may be attributed to certain phytochemicals such as flavonoids, saponins, terpenoids, antioxidants and *α*-amylase inhibitory activity, as supported by various studies.

Several studies on different parts of *S. pinnata* have shown promising hypoglycemic and antidiabetic activities. It has been reported that methanol extract of the bark of *S. pinnata* produced significant antidiabetic activity in alloxan-induced diabetic rats [16]. The methanol extract of *S. pinnata* fruits exhibited a mild antidiabetic effect on alloxan-induced diabetic rats [17]. Another species of the genus *Spondias,* i.e., *S. mombin* leaves, possesses significant antidiabetic and antioxidant properties [39, 40]. Some potent secondary metabolites including flavonoids, phenolic acids, tannins, and triterpenes have been identified from *S. mombin* leaves [39–41]. Attanayake et al., [42] reported significant antihyperlipidemic and antihyperglycemic effects of aqueous bark extract of *S. pinnata* in streptozotocin-induced diabetic rats. The bark extract was able to induce *β*-cell regeneration in the pancreas of diabetic rats, which in turn increased the biosynthesis of insulin and improved glucose tolerance in *S. pinnata*-treated diabetic rats [42]. Likewise, the aqueous extracts of *S. pinnata* roots showed hypoglycemic activity with a significant decrease in blood glucose levels after four hours of treatment compared to glibenclamide in the oral glucose tolerance test [43]. Furthermore, our study on the hypoglycemic potential of leaves extract of *S. pinnata* has provided scientific evidence on the traditional use of the leaves for diabetes, and it is also the first *in vivo* hypoglycemic study of the leaves extract to our best knowledge.

## 5. Conclusion

Medicinal herbs have been used for the treatment and control of diabetes across the globe. *S. pinnata* leaves extract exhibited significant hypoglycemic activity in the animal model. The phytochemical constituents such as flavonoids, phenolics, saponins, and terpenoids might be the possible reason for its hypoglycemic action. Therefore, *S. pinnata* leaves may be a promising source of new antihyperglycemic agents, and a detailed mechanism-based study and isolation of bioactive compounds from *S. pinnata* would be beneficial in the future.

## Figures and Tables

**Table 1:** Phytochemical screening of leaves extract of *S. pinnata*.

Test	Interferences
Alkaloids	
(a) Mayer's test	−
(b) Hager's test	−
(c) Wagner's test	−
Glycosides (modified Borntrager's test)	−
Saponins (foam test)	+
Phenols (ferric chloride test)	+
Flavonoids (alkaline reagent test)	+
Tannin (gelatin test)	−
Terpenoids (Salkowski test)	+

+: presence; −: absence.

**Table 2:** Effect of *S. pinnata* leaves extract on fasting blood glucose level in normal rats.

Group (*n* = 5)	Dose (mg/kg)	Average blood glucose level (mg/dl)				
		0 h	0.5 h	1 h	2 h	3 h
Control		102.6 ± 10.66	105.8 ± 13.08	103.6 ± 6.22	100.2 ± 7.15	98.8 ± 6.76
*S. pinnata*	250	94.6 ± 6.02	99.4 ± 3.64	99.0 ± 10.09	96.6 ± 5.54	91.0 ± 8.71
*S. pinnata*	500	95.8 ± 3.63	105.0 ± 4.35	90.2 ± 10.25^*∗*^	84.2 ± 10.13^*∗*^	79.2 ± 5.40^*∗*^
Metformin	100	96.4 ± 3.04	98.4 ± 6.18	86.2 ± 3.56^*∗*^	78.2 ± 4.49^*∗*^	69.2 ± 3.70^*∗*^

Values are expressed as mean ± SD, (*n* = 5). ^*∗*^Statistically significant when compared to the control group at *p* < 0.05 (ANOVA followed by Dunnett's post hoc test).

**Table 3:** Effect of *S. pinnata* leaves extract on blood glucose level in glucose-induced hyperglycemic rats (OGTT).

Group (*n* = 5)	Dose (mg/kg)	Average blood glucose level (mg/dl)				
		0 h	0.5 h	1 h	2 h	3 h
Control		98.6 ± 7.50	148.8 ± 10.84	120.6 ± 12.77	107.0 ± 9.92	100.2 ± 7.56
*S. pinnata*	250	92.8 ± 4.20	112.4 ± 8.79^*∗*^	101.6 ± 9.31^*∗*^	95.8 ± 8.34^*∗*^	90.6 ± 5.81
*S. pinnata*	500	96.0 ± 5.24	124.4 ± 8.17^*∗*^	103.4 ± 7.05^*∗*^	94.8 ± 4.14^*∗*^	82.8 ± 4.43^*∗*^
Metformin	100	95.8 ± 4.20	127.8 ± 8.16^*∗*^	103.4 ± 6.46^*∗*^	89.0 ± 6.51^*∗*^	76.0 ± 8.68^*∗*^

Values are expressed as mean ± SD, (*n* = 5). ^*∗*^Statistically significant when compared to the control group at *p* < 0.05 (ANOVA followed by Dunnett's post hoc test).

## Data Availability

The data used to support the findings of this study are available from the corresponding author upon request.
